# Supplementary material for: “I was screaming hallelujah”: Patient and provider perceptions of blood-based testing for colorectal cancer screening
Source: PLoS One. 2023 Dec 21;18(12):e0295685. doi: 10.1371/journal.pone.0295685 (PMC10734920; doi:10.1371/journal.pone.0295685)
Supplement: S1 File — (DOCX) [file pone.0295685.s003.docx]

**Guardant Health Patient Interview Guide**

***Questions for Participants that Complete Blood Test***

**Introductory/background**

1. How long have you been a member of Kaiser Permanente?
2. Prior to joining this study, had your provider or someone ever recommend you screen for colon cancer? Please describe.
   1. Who suggested this?
   2. What was your reaction or response?
   3. Prior to this study, had you ever screened for colon cancer before (probe on if ever completed a stool-based kit or colonoscopy in past with Kaiser or another system)?
   4. [If have screened previously]: What motivated you to do so?
   5. [If have not screened previously]: What got in the way of following thru on the screening recommendation?

**Recent FIT Offering**

1. A few months back you may have been offered a colon cancer screening stool test, also called a FIT, by Kaiser – do you recall your reaction to being offered that screening test?
   1. Was this the first time being offered a FIT for colon cancer screening?
   2. How did you receive it – by provider in-clinic? By mail?
   3. What did you decide to do and why?
   4. What got in the way of completing the FIT?
   5. Did you have any follow up conversation with your provider or anyone else regarding completing the FIT or not?
   6. What might have motivated you to complete the FIT?

**Blood test – Guardant SHIELD COLON CANCER screening test**

*Our study team reached out to you as someone who did not return their FIT test recently, to offer you a blood test instead that can find signs of colorectal tumors in a person’s blood.*

1. As best you can recall, what was your reaction to the initial outreach inviting you to join the study and complete the blood test as a colon cancer screening option?
2. What motivated you to complete the blood test?
   1. What helped you to feel open and confident to try this blood test option? (ease of appointment, familiar with blood draws, trustworthy option since DNA related test, less ‘yuk’ factor than a FIT, curiosity, only complete it every 3 years so don’t have to do it as frequently, etc.)
   2. What concerns or worries did you have about the blood test option? (accuracy of result, side effects from blood draw, don’t like needles, results, having to attend another appointment/transportation, other?)
3. What was your experience like in completing the blood test? Please describe
   1. What made this process work well for you? (including before, during, and right after blood draw)
   2. What made this process challenging or concerning for you? (including before, during, and right after blood draw)
4. How did you receive your results from the blood test? How long did it take to receive them?
   1. Was this acceptable to you
   2. Was the outcome of the blood test clear to you? Did you have any questions or concerns about the results?
   3. Did anyone, your provider or anyone else, speak to you about your result? Please describe.
   4. For abnormals: Was it clear to you a follow-up colonoscopy would be the next step? Did anyone reach out to you about scheduling a follow-up colonoscopy? How did this go? What did you decide to do and why?
5. What made completing the blood test as a screening tool for colon cancer more appealing than completing a FIT? (*probe on reasons: because free? Part of study? Less “ick”? etc*)
   1. What made completing the blood test as a screening tool less appealing than completing a FIT?
6. If suggested by your provider or Kaiser (letter or email portal) to complete the blood test again in the future for colon cancer screening, would you complete it again? Why or why not?
   1. The blood test is recommended every 3 years (versus a FIT yearly), how do you feel about completing it every 3 years?
   2. How much would you be willing to pay for the blood test as a colon cancer screening tool in the future (e.g., *typical OOP cost between $25 to $40 and possible full cost to patient $400 to $600, probe on patient expectation re cost*)
   3. If given a choice between the blood test and a FIT for colon cancer screening, which one would you choose for future screening? Why is that? (*probe on tradeoffs between yearly/less costly FIT can do at home but requires dealing with poop versus going every 3 years for a blood draw that could cost several hundred dollars*)

**Advice/Future Implementation**

1. How do you feel about Kaiser possibly offering this blood test as a colon cancer screening option? Is it something they should offer, why or why not?
2. If Kaiser were to offer the blood test regularly, how might you prefer to learn or know about it?
   1. Provider recommendation – in-person visit
   2. Provider recommendation – letter, text, or email portal communication
   3. General Kaiser outreach – letter, call, text, email portal
   4. Offered at another laboratory or in-clinic appointment
3. What advice or suggestions do you have regarding educating or motivating patients to consider colon cancer screening using this blood test option? Using the FIT option?
4. What advice or suggestions do you have regarding improving the process of getting the blood test? Of receiving the results?
5. Is there anything else you would like to share or you feel is important for us to know regarding your decision to get the blood test or your experience with obtaining it?

***Questions for Participants that Booked but No-showed to Complete Blood Test***

**Introductory/background**

1. How long have you been a member of Kaiser Permanente?
2. Prior to joining this study, had your provider or someone ever recommend you screen for colon cancer? Please describe.
   1. Who suggested this?
   2. What was your reaction or response?
   3. Prior to this study, had you ever screened for colon cancer before (probe on if ever completed a stool based kit or colonoscopy in past with Kaiser or another system)?
   4. [If have screened previously]: What motivated you to do so?
   5. [If have not screened previously]: What got in the way of following thru on the screening recommendation?

**Recent FIT Offering**

1. A few months back you may have been offered a colon cancer screening stool test, also called a FIT, by Kaiser – do you recall your reaction to being offered that screening test?
   1. Was this the first time being offered a FIT for colon cancer screening?
   2. How did you receive it – by provider in-clinic? By mail?
   3. What did you decide to do and why?
   4. What got in the way of completing the FIT?
   5. Did you have any follow up conversation with your provider or anyone else regarding completing the FIT or not?
   6. What might have motivated you to complete the FIT?

**Blood test – Guardant SHIELD COLON CANCER screening test**

*Our study reached out to you as someone who did not return their FIT test recently, to offer you a blood test instead that can find signs of colorectal tumors in a person’s blood.*

1. As best you can recall, what was your reaction to the initial outreach inviting you to join the study and complete the blood test as a colon cancer screening option?
2. What were your reasons to not complete the blood test to screen for colon cancer?
   1. What concerns or worries did you have about the blood test option? (accuracy of result, side effects from blood draw, dislike needles or blood draws, results, having to attend another appointment/transportation, possible costs, other?)
3. What might have helped you to consider completing the blood test for colon cancer screening?
   1. Probe on: more information on blood test; direct recommendation from provider; how accurate or not the test is; more explanation on how often it is required or what happens with the results; more information on cost of test to patient, other?
4. Is the idea of completing a blood test for colon cancer screening more or less appealing to you than completing a stool cared test such as a FIT? Please describe.
   1. The blood test is recommended every 3 years (versus a FIT yearly), how do you feel about the idea of completing it every 3 years?
   2. How much would you be willing to pay for the blood test as a colon cancer screening tool in the future (e.g., *typical OOP cost between $25 to $40 and possible full cost to patient $400 to $600, probe on patient expectation re cost*)
   3. If given a choice between the blood test and a FIT for colon cancer screening, which one would you choose for future screening? Why is that? What barriers or challenges still exist for you? (*probe on tradeoffs between yearly/less costly FIT can do at home but requires dealing with poop versus going every 3 years for a blood draw that could cost several hundred dollars*)

**Advice/Future Implementation**

1. How do you feel about Kaiser possibly offering this blood test as a colon cancer screening option? Is it something they should offer, why or why not?
2. If Kaiser were to offer the blood test regularly, how might you prefer to learn or know about it?
   1. Provider recommendation – in-person visit
   2. Provider recommendation – letter, text, or email portal communication
   3. General Kaiser outreach – letter, call, text, email portal
   4. Offered at another laboratory or in-clinic appointment
3. What advice or suggestions do you have regarding educating or motivating patients to consider colon cancer screening using this blood test option? Using the FIT option?
4. Is there anything else you would like to share or you feel is important for us to know regarding your decision to not get the blood test for colon cancer screening?

**Guardant Health Provider/Specialist Interview Guide**

**Introduction and general questions for PCPs or GIs/specialists**

1. How familiar are you with blood-based tests that looks for signals of CRC in blood? [*Whether familiar or not offer some of data from text below and reference the snapshot before moving on]*
   1. How have you heard or learned about it / discussed among colleagues?

*Shield is a test that uses various laboratory methods to look for signals of colorectal cancer in your blood. (The test looks for DNA shed by tumors and other changes in the proteins in your blood).*

*Early data shows strong performance in detecting cancer (it will find more than 9 of 10 cases of cancer), however, the performance is low in detecting large polyps (i.e., advanced adenomas). Cancer and large polyp detection of the blood-based test is on par with other non-invasive CRC screening tests (e.g., stool tests) [show visual snapshot as well]. The blood test is recommended every 3 years. Patients who have an abnormal test result should get a colonoscopy. Patients who have a normal colonoscopy result following an abnormal Shield test result are considered at average risk for colorectal cancer, and should get regular screening, using any recommended test (stool-test, colonoscopy, etc.).*

1. What are your thoughts or reactions to it as a CRC screening option? Probe for advantages and disadvantages
   1. How would you compare it as a screening option to yearly FIT? (*accuracy, frequency, convenience, costs, etc*)
   2. What are your primary concerns, if any, for utilizing it as a CRC screening tool? (*accuracy, cost to patient, cost to health system, other*)
2. How do you think patients might react to it as a screening option?
3. [*or if PCP has a ppt in study*]: What is your reaction to receiving the results of the blood test for your patients?
   1. Does the result make sense? Any question or concerns re the result?
   2. How do your patients respond or react to the result? Any questions or concerns being expressed?
   3. If an abnormal result, are patients willing to follow through on a colonoscopy?
4. If kaiser were to offer the blood test as a screening option, how would you like to see it communicated and implemented?
   1. How do you see this working within the current screening program of offering FIT?
   2. What benefits do you see in making the blood test an option along with FIT?
   3. What downside do you anticipate?
5. What more would you like to know or understand about it as a screening option?
6. Anything else?

**Additional questions regarding specialist tracking results (ask along with above questions)**

1. What are your thoughts/reactions to the abnormal results from the Guardant SHIELD blood test?
   1. Any concerns about results/confidence in results?
   2. What are patient reactions to the results? Are results understandable to patients?
   3. Do patients seem to trust the result or not?
   4. Are patients willing to follow through on their colonoscopy from the abnormal finding?
   5. How our PCPs of patients with abnormal results reacting? What questions or concerns are arising from these PCPs?
2. How might the result process be improved?
   1. Improved for specialists/clinicians
   2. Improved for patients
3. How might your answers to these questions differ, if at all, if the abnormal screening result came from a stool-based test versus the blood-based test? [*Interviewer note:* probe on if how they treat a blood-based test different or similar to a stool-based test]
