# Supplementary material for: “I was screaming hallelujah”: Patient and provider perceptions of blood-based testing for colorectal cancer screening
Source: PLoS One. 2023 Dec 21;18(12):e0295685. doi: 10.1371/journal.pone.0295685 (PMC10734920; doi:10.1371/journal.pone.0295685)

**Snapshot overview for blood-based DNA tests that screen for CRC – shared during Provider interviews**

- Blood-based colorectal cancer (CRC) screening tests may improve adherence to screening guidelines.
- Blood-based tests use various laboratory methods to look for signals of colorectal cancer in your blood. (The test looks for DNA shed by tumors and other changes in the proteins in your blood).
- Early data shows strong performance in detecting cancer (it will find more than 9 of 10 cases of cancer), however, the performance is low in detecting large polyps (i.e., advanced adenomas). Cancer and large polyp detection of the blood-based test is on par with other non-invasive CRC screening tests (e.g., stool tests).
- The blood test is recommended every 3 years.
- Blood tests are not included as an A or B recommendation by national organizations. Thus, out of pocket cost to patients approximately $400-600.
- Patients who have an abnormal test result should get a diagnostic colonoscopy.
- Patients who have a normal colonoscopy result following an abnormal blood-based screening result are considered at average risk for colorectal cancer No additional follow-up tests are recommended for these patients. They can continue to screen using any available option (stool-test, colonoscopy, or blood test if it is available).


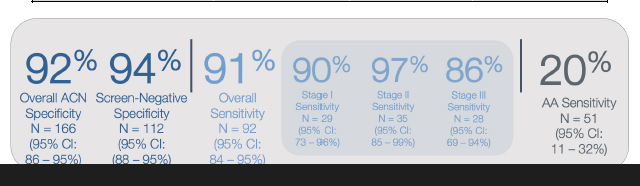

Supplement: S2 File — (DOCX) [file pone.0295685.s004.docx]
