# Supplementary material for: “I was screaming hallelujah”: Patient and provider perceptions of blood-based testing for colorectal cancer screening
Source: PLoS One. 2023 Dec 21;18(12):e0295685. doi: 10.1371/journal.pone.0295685 (PMC10734920; doi:10.1371/journal.pone.0295685)
Supplement: S3 File — (DOCX) [file pone.0295685.s005.docx]

**Examples of code templates for patient and provider interviews**

*Templates developed from interview guide structure and content from multiple reviews of transcripts. Each interview is then read multiple times and relevant information from transcript is extracted into topical code area along with any illustrative quotes*.

**Patients**

| **Topical code area** | **Extracted data from transcript (with participant ID)** | **Relevant quotes** |
| --- | --- | --- |
| **Years as health system member** |  |  |
| **Prior health system CRC* screening offers** |  |  |
| **Reactions to prior CRC screening offers** |  |  |
| **Reasons to decline past screening or barriers to FIT**** |  |  |
| **Initial reaction to blood test offer** |  |  |
| **Appeal / motivation to complete blood test** |  |  |
| **Reasons to not complete blood test** |  |  |
| **Questions or concerns about blood test** |  |  |
| **General experience of completing blood test** (barriers / facilitators) |  |  |
| **Experience receiving blood test results** |  |  |
| **Discussion with providers regarding blood test results** |  |  |
| **Future actions: choice between FIT and blood test option** |  |  |
| **Acceptability of health system offering blood test option in future** |  |  |
| **Other advice or improvements** |  |  |

**Providers**

| **Topical code area** | **Extracted data from transcript (with participant ID)** | **Relevant quotes** |
| --- | --- | --- |
| **Background** (e.g. type of provider, CRC screening role) |  |  |
| **Prior awareness of blood-based CRC* screening options** |  |  |
| **Experiences offering FIT**** (barriers / facilitators) |  |  |
| **Advantages of blood test option** |  |  |
| **Disadvantages of blood test option** (e.g. concerns) |  |  |
| **Questions about blood test option** |  |  |
| **Experience delivering blood test result** |  |  |
| **Future actions: choice between FIT and blood test option** |  |  |
| **Acceptability of health system offering blood test option in future** |  |  |
| **Other advice or improvements** |  |  |

*CRC=Colorectal cancer; **FIT=Fecal immunochemical test
