# Supplementary material for: “I was screaming hallelujah”: Patient and provider perceptions of blood-based testing for colorectal cancer screening
Source: PLoS One. 2023 Dec 21;18(12):e0295685. doi: 10.1371/journal.pone.0295685 (PMC10734920; doi:10.1371/journal.pone.0295685)
Supplement: Attachment — Submitted filename: PLOS ONE Review8.14.2023.docx [file pone.0295685.s006.docx]

PLOS ONE Review:

This qualitative study assesses perceptions of blood-based colorectal cancer screening modality among patients who did not previously complete/adhere to a stool-based screening test. Perceptions of several types of health providers are also assessed. This is a well-written manuscript and overall, the findings are interesting, and I believe have important implications for increasing screening and patient comfort/satisfaction with information about screening. A more robust background on the blood test is needed to orient the reader and several implications of the findings can be expanded on in the discussion. Details comments are below.

Introduction:

- Authors state that blood-based testing for cell-free DNA is the newest option. It would be helpful to know the general availability dates of FIT tests and blood tests for context. What year was blood-based approved? What type of USPTF rating does it have? Given that the authors note that the commercially available blood test that was used was not approved by the FDA at the time of the study, some additional rationale for the use of the blood-based test is needed. Also, is it currently FDA-approved, and on what date did this occur?
- Authors note that participants “passively declined” a FIT test. It is unclear what this exactly means. Perhaps more detail in the methods would help to understand the use of this term.

Methods:

- Some of the detailed information about the Shield blood test in the Methods section may be more appropriate in the background section. Perhaps add this as a comparison to the FIT test since this comparison is made as part of the rationale.
- The statement that patients were notified that the test is not validated for stand-alone use in diagnosis is a bit misleading without additional context. For example, does this mean that the full protocol for a positive on this test includes a follow-up test? If so, adding that context would clarify for the reader. Also, this is the same protocol for FIT (positives are followed up with a colonoscopy, so again, this may be information more suitable in the introduction section to set the reader up to understand the nuances of each type of test (The FIT, which was declined, and the subsequent blood-test).
- What input of the research team informed semi-structured guides? Also, how was the literature used to inform guides? Were items adapted from similar types of studies or were themes used to develop new questions? Also, was a code book used for coding and was the data managed with any specific tools? (Excel, Nvivo, other?) In general, I may be helpful to review a qualitative checklist to ensure all details are included (SRQR or COREQ checklists).
- Also, were interviews recorded? Transcribed? There is mention to showing a visual to providers during interviews, but not clear how that was conducted if interviews were conducted via phone, were visuals emailed to providers prior to the interview?
- What was the rationale for interviewing patients who did and did not complete the blood-based test? Were there specific accrual goals for these groups, and were there plans to compare responses? If only interested in those who did complete the blood tests, why did you interview those who did not complete them? (I see that later in the results it indicates that those 4 were scheduled to complete it, perhaps that can be clarified in the abstract and methods), The way they are discussed sets them up as different groups of participants, when maybe it's more accurate to think of them as being interviewed at an earlier stage in the process of screening. Do you know if they eventually went on to complete the screening via blood? That would then give a bit more confidence that they are a homogenous group (e.g., declined FIT but completed or scheduled to complete a blood-based test).
- In the description of the participants is there a way to provide geographic information? For example, are participants in urban vs. rural areas? This may impact perceptions of a screening modality that requires in-person access vs. one that can be completed remotely.

Results:

- One concern with the results is that authors report that most (14/15) participants said they would choose blood tests over FIT, however, since most of the participants were already people who had declined fit and completed blood tests. I would just be careful not to indicate that blood tests are the preferred modality overall, b/c this specific population was already possibly in a position to prefer a different modality since they had already declined a FIT. Maybe even in the discussion, looking into if there are certain groups who may prefer blood over stool and if there are implications for screening campaigns using audience segmentation. Etc.
- I thought it interesting that providers were concerned about cost, but patient participants did not list that as a concern. What was the income range of participants?

Discussion:

- I thought it was interesting that participants viewed blood as more accurate than stool. Could authors provide some comparison of accuracy between tests in the intro or discussion?
- Also, was interesting that having the blood test scheduled was a perceived benefit for participants. Have there been any studies where FIT tests were tied to appointments or “due dates”? what are the implications for increasing screening rates based on some of these identified findings?
